# Biointegration of a partially decellularized tracheal scaffold in a porcine model - preliminary results

**DOI:** 10.1038/s41598-026-37823-1

**Published:** 2026-02-21

**Authors:** Augustin Vigouroux, Yannis Bonnin, Nicolas Gendron, Sabrina Kellouche, Rémy Agniel, Patrick Bruneval, Daniel Balvay, Jean-Marc Allain, Stéphanie Chhun, Hervé Kempf, Romain Hugon, Magali Devriese, Caroline Sansac, Jérôme Larghero, Lousineh Arakelian, Briac Thierry

**Affiliations:** 1 Université Paris Cité, Inserm, IRSL, U1342, CIC-BT CBT501, Paris, F-75475 France; 2 Department of Hematology, Assistance Publique Hôpitaux de Paris-Centre Université de Paris (APHP-CUP), Hôpital européen Georges Pompidou, Paris, F-75015 France; 3 Université Paris Cité, INSERM UMR-S 970, Paris Cardiovascular Research Centre, Paris, France; 4 CY Cergy Paris Université, Institut des Matériaux, I‐MAT FD4122, Equipe de Recherche sur les Relations Matrice Extracellulaire‐Cellules, ERRMECe EA1391, Cergy, F-95000 France; 5 Department of Pathology, AP-HP, Georges Pompidou European Hospital, Paris, F-75015 France; 6 Université Paris Cité, Inserm, PARCC, U970, Paris, F-75015 France; 7 Laboratoire de mécanique des solides, CNRS, École polytechnique, Institut Polytechnique de Paris, Palaiseau, France; 8 Inria, Palaiseau, F-91120 France; 9 Institut Necker Enfants-Malades (INEM), INSERM U1151, Paris, F-75015 France; 10 Laboratoire D’Immunologie Biologique, Hôpital Universitaire Necker-Enfants Malades, AP-HP, Paris, F-75015 France; 11 IMoPA, UMR 7365 CNRS-Université de Lorraine, Vandœuvre-lès-Nancy, France; 12 Laboratoire d’Immunologie et Histocompatibilité, Hôpital St‐Louis, AP‐HP, Paris, F-75010 France; 13 Banque de Tissus Humains, Hôpital St‐Louis, AP‐HP, Paris, F-75010 France; 14 Unité de Thérapie Cellulaire, AP-HP, Hôpital Saint-Louis, Paris, France; 15 Department of Pediatric Otolaryngology-Head and Neck Surgery, AP-HP, Hôpital Universitaire Necker – Enfants Malades, 149, rue de Sèvres, Paris, F-75015 France

**Keywords:** Tracheal replacement, Decellularization, Biointegration, Immunosuppression, Vascularization, Diseases, Medical research

## Abstract

Some pediatric tracheal pathologies remain therapeutic dead ends for which current palliative strategies are fraught with serious complications. With the aim of a tracheal replacement, our team has previously developed and patented a clinical grade partially decellularized trachea (PDT) from porcine tracheas. The aim of this work was to study and compare the biointegration mechanisms of this PDT *in vivo*, in a pig cervical muscle, with or without immunosuppressant. The secondary objective was to evaluate the optimal maturation time of the PDT in this heterotopic position. In total, 11 female Large White/Landrace pigs, weighing between 50 and 70 kg were included in this study. The mean age of the animals at the implantation was 4.8 months. The PDTs were implanted in a cervical muscle of pigs for either 28 days, with or without cyclosporin A treatment, or for 56 days without immunosuppression. Histological evaluation showed very good PDT biointegration, characterized by neovascularization and fibroblast colonization, and no detectabale infection. Additionally, tissue and blood analyses showed no signs of graft rejection or surrounding tissue necrosis. Immunosuppression did not show any superiority in terms of biointegration after 28 days of treatment. After 56 days of implantation, a more significant degradation of the cartilage. Therefore, the optimal condition for PDT maturation proved to be 28 days, without immunosuppression.

**Supplementary Information:**

The online version contains supplementary material available at 10.1038/s41598-026-37823-1.

## Introduction

In pediatrics, certain rare tracheal diseases remain incurable due to the absence of both medical treatment and a suitable tracheal substitute. Furthermore, there is no standardized surgical technique for tracheal replacement. Patients requiring extensive tracheal resection are currently managed with palliative care and ultimately succumb to their tracheal illness^1^.

For these patients, the surgical challenge of tracheal replacement lies in ensuring a long-term airway patency in a growing child with a limited thoracic volume, often in emergency conditions. Circumferential tracheal replacement represents a potential therapeutic alternative, provided that an adequate tracheal substitute is available.

A viable tracheal substitute requires multiple criteria: it must be non-immunogenic, biocompatible, and vascularized. Its luminal epithelial lining should serve as a barrier to the external environment, while withstanding the mechanical constraints imposed by breathing and cervical mobility^2^. In pediatric applications, it must also have a limited steric encumbrance and ideally provide a sufficient respiratory caliber, without requiring endoluminal stenting. Different substitutes and technics have been used in the literature including synthetic materials, thyrotracheal transplantation, aortic grafts, autologous tissue contructs and tissue engineering^3^. However, they present a number of limits. For instance, synthetic materials do not biointegrate, and thyrotracheal transplantation requires lifetime immunosuppression, not recommended for children. To overcome these problems, we chose tissue engineering and decellularizatio for developing a tracheal substitute.

Organ decellularization has emerged as a promising field of research. This technique aims to reduce the immunogenicity of a donor tissue or organ by eliminating its cells and genetic material, thereby minimizing the risk of graft rejection, without the need of immunosuppressive therapy^4^. Several decellularization protocols have been developed for the trachea, utilizing a range of physical, enzymatic, and chemical treatments^5^.

However, tracheal decellularization presents unique challenges due to its composition of soft tissue and rigid cartilage rings. Complete decellularization risks significantly compromising the biomechanical properties of the cartilage, leading to tracheal malacia and potential post-implantation respiratory complications. To mitigate these risks, the concept of partial decellularization has been introduced. This approach involves shorter treatments that selectively remove the highly immunogenic mucosal and submucosal compartments while preserving the structural integrity of the tracheal cartilage^6^.

Previously, it was shown that in a mouse model, partially decellularized mouse tracheas retained cartilage immune privilege and a less cartilage degradation and T-cell infiltration, compared to native tracheal allografts^7^.

The successful *in vivo* functionality of a partially decellularized trachea (PDT) depends on its neovascularization by the recipient organism, ensuring an adequate supply of nutrients and oxygen for cellular recolonization, as well as protection from infection^8^. To facilitate this process, an initial phase of *in vivo* pre-maturation in a heterotopic position is essential before orthotopic tracheal replacement. The local and systemic immunological responses that occur during this biointegration phase remain an active area of research.

For a preclinical study, selecting an appropriate large-animal model is crucial. The pig is particularly well-suited for this purpose, given its tracheal anatomy, which closely resembles that of humans, and its well-established immunological similarity^9^. Despite advances in the field, no studies have yet investigated the impact of immunosuppressive treatment on local and systemic immune responses to a decellularized trachea *in vivo* in a porcine model.

## Current study and objectives

Recently, our team has developed and patented a clinical-grade PDT production protocol using porcine tracheas^10^ (European Patent No. EP23306699,2). In this study, we evaluated the implantation of these PDTs into a cervical muscle of pigs for up to 56 days.

The primary objective was to compare the *in vivo* biointegration and vascularization of PDT with and without immunosuppressive treatment. The secondary objective was to determine the optimal maturation time of the PDT prior to its orthotopic tracheal replacement.

## Results

### Sterility maintenance of partially decellularized tracheas from decontamination to implantation

For the four PDT samples analyzed at each step of the decellularization, and before implantation, the bacterial and fungal samples cultures were negative after decontamination with antibiotics. Sterility was maintained until the end of the PDT fabrication process (Table 1).

**Table 1:** Results of the microbiological study analysis (aerobic and anaerobic bacteriological bacterial cultures, mycological fungal cultures) of samples from 4 tracheas during a partial decellularization protocol.

**Trachea**	**After decontamination**	**After decellularization**	**Before cryopreservation**
**# 1**	Negative	Negative	Negative
**# 2**	Negative	Negative	Negative
**# 3**	Negative	Negative	Negative
**# 4**	Negative	Negative	Negative

### Animal cohorts and surgical procedures for PDT implantation

Between June 2023 and June 2024, eleven pigs were implanted with the PDT, monitored and subsequently explanted. The PDT was placed either in the sterno-cephalic muscle (*n* = 6) or in the sterno-hyoid muscle (*n* = 5). The mean age of the animals at the implantation point was 4.8 months (± 0.75). Among these animals, five pigs received no immunosuppressive treatment (IS^−^ group), four pigs were treated with cyclosporin A (IS^+^ group); both groups had the PDT implanted for 28 days. Additionally, two pigs, without immunosuppressive treatment, were implanted for an extended period of 56 days.

General clinical condition of the animals.

No general complications were observed during the monitoring period, regardless of the treatment group. All animals remained afebrile, exhibited normal behavior, and maintained a regular and satisfactory feeding pattern.

**Fig. 1:**
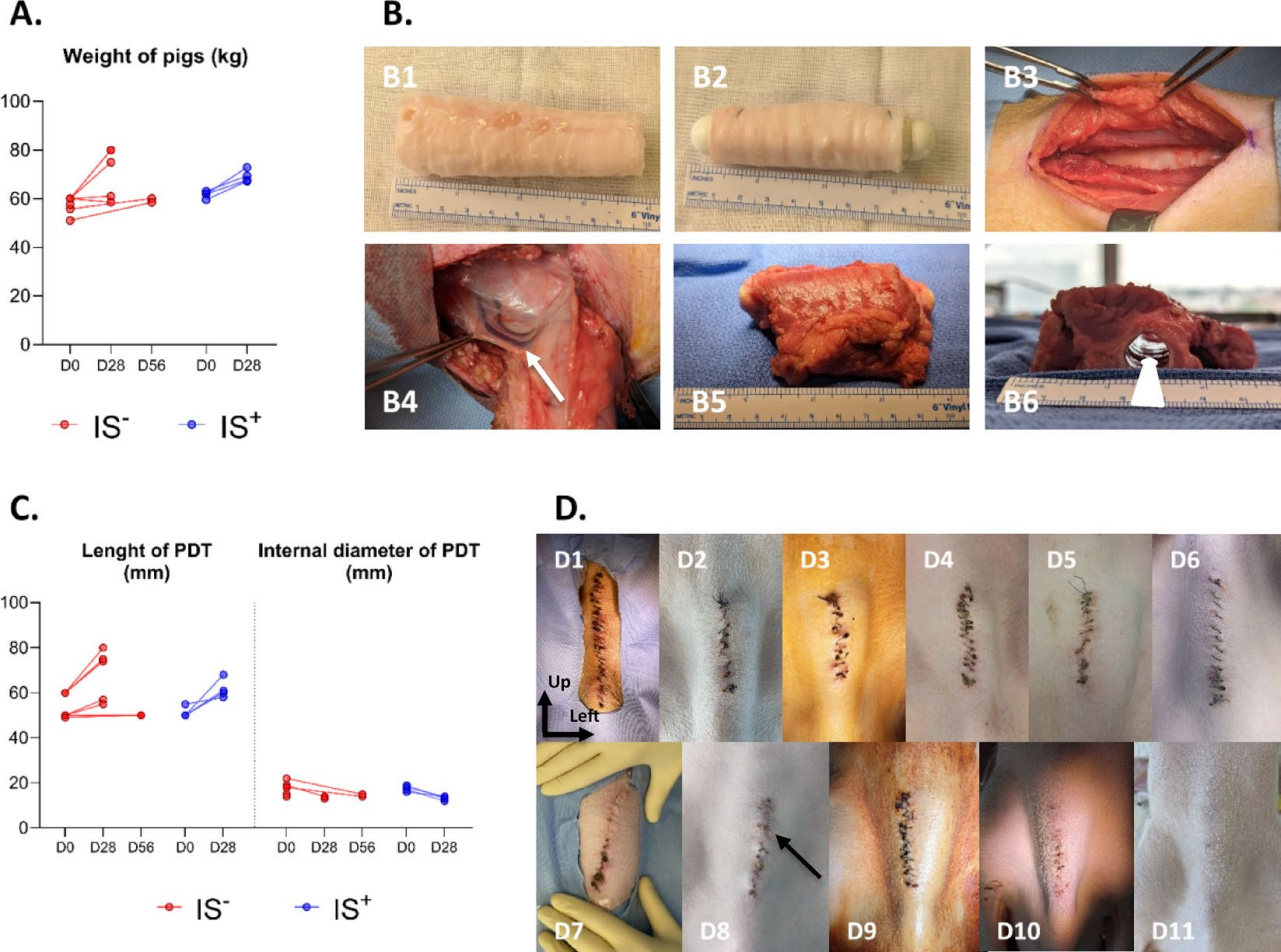
A. Evolution of the animals’ weight between implantation (D0) and explantation (D28 or D56), in the two treatment groups (without immunosuppressant IS^-^ or with immunosuppressant IS+). **B.** Macroscopic views of the surgical protocol. The top row shows a partially decellularized trachea (PDT) after cryopreservation and thawing (B1), extension on a silicone stent (B2) and placement in the cervical muscle compartment (B3). The bottom row shows the operative results during explantation: perforating vascular pedicles directed towards the recipient muscle and PDT (B4), healthy-looking muscle-PDT complex (B5) and PDT with a wide-open lumen after stent removal (B6); the white arrow illustrates the preservation of a tracheal diameter without collapse under the effect of gravity alone. **C.** Evolution of PDT length and internal diameter (before stent extension and after stent removal) between implantation (D0) and explantation (D28 or D56), in the two treatment groups (IS- or IS+). **D.** Scar status on the day of explantation, in five pigs from the D28 IS^-^ group (D1 to D5), the four pigs from the IS^+^ group (D6 to D9), and the two pigs IS^-^ explanted at D56 (D10 and D11). There were no significant local complications, apart from a superficial subcentimetric abscess in one IS^+^ pig (black arrow). At D56, healing was satisfactory.

During PDT maturation, the median weight gain was + 15 kg [−0.2;+20] in 28 days for the IS^−^ group, and + 7 kg [+ 5.8;10.1] in 28 days for the IS^+^ group, showing no significant difference in weight evolution between groups (Wilcoxon signed-rank test, *p* = 0.375). Concerning the two animals sacrificed at D56, the median weight gain was + 5.9 kg [+ 4.4;+7.5] (Fig. 1*A*).

One pig in the IS^+^ group experienced vomiting during anesthetic induction prior to implantation, with suspected aspiration. As a precaution, it received probabilistic antibiotic treatment with Amoxicillin (Vetrimoxin 750 mg every 48 h) for one week. No subsequent general or respiratory infections were observed.

### Loco-regional response

#### Surgical outcomes and PDT integrity

No major complications, such as hematoma, scar dehiscence, or deep surgical site infection, were observed at the surgical site, in any of the 11 animals. The average skin incision length at implantation was 10.7 cm (± 2.74), ranging from 14.5 cm in the first animals to 7 cm in the later ones.

Before implantation, the cryopreserved and thawed PDTs remained intact, with no major visible damage (Fig. 1*B1*). To prevent liquid accumulation inside the PDT, a closed stent was placed inside the lumen. Th PDT was easily extended and secured onto the stent without tearing (Fig. 1*B2*). Implantation within the muscle (Fig. 1*B3*) was performed without difficulty.

At explantation, following excision of the muscle-PDT complex with clear visualization of the lateral perforating arteriovenous pedicles supplying the surrounding muscle (Fig. 1*B4*), the recipient muscle displayed satisfactory healing, good viability, and adequate vascularization (Fig. 1*B5*). No PDT displacement was observed. After stent removal, the lumen remained fully open macroscopically, without collapsing (Fig. 1*B6*,* white arrow*).

#### Cartilage integrity and tracheal harvesting

Transverse sectioning of the PDT revealed spontaneous cartilage cleavage throughout its thickness in both treatment groups. The tracheal surgical approach for native trachea harvesting was straightforward and not hindered by the minimal dissection, performed during the initial procedure.

### Macroscopic characterization of PDT dimensions: length and diameter

In all animals implanted for 28 days, PDT length increased after maturation and stent removal: the median increase was + 11 mm [+ 6;+16.5], ranging from + 3 to + 20 mm. Conversely, for animals implanted for 56 days, the median evolution of PDT length was + 0.5 mm [0;+1] (Fig. 1*C*). As detailed in Table 2, at D56, the PDT was found disinserted from one of the stent ends during explantation in one animal.

The internal diameter of PDTs decreased between implantation (before stenting) and explantation (after stent removal) in all animals. The median decrease was − 5.0 mm [−5.5;−3] at D28, and − 5.5 mm [−7;−4] at D56 (Fig. 1*C*).

#### Post-explantation observations

At explantation, cervical scars were clean and non-inflammatory (Fig. 1*D*), except in one immunosuppressed pig, which developed an inflamed scar with a minor skin abscess (Fig. 1*D8*,* black arrow*). The abscess was limited to the skin, with no subcutaneous collection.

##### Histological assessment of biointegration and structural integrity of PDTs before implantation

**Fig. 2:**
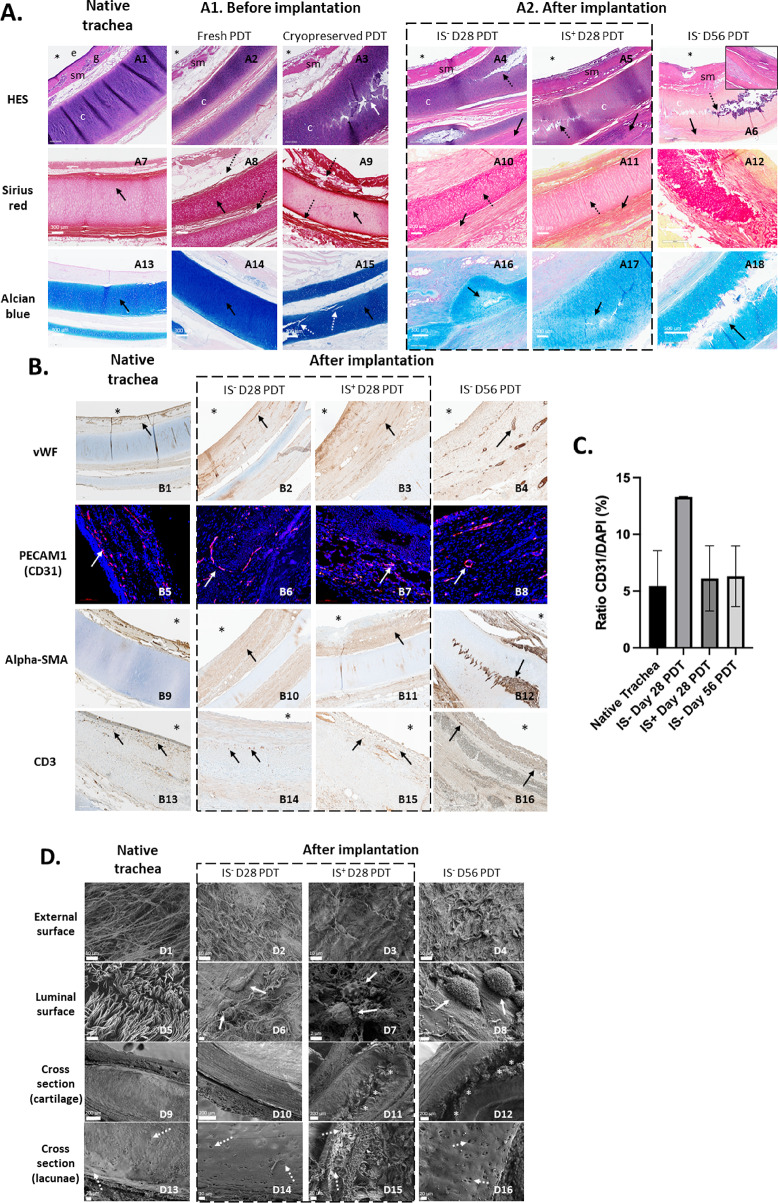
**(A)** Axial histological sections using light microscopy with Hematoxylin-Eosin-Safran (HES), Sirius red and Alcian blue staining: native porcine trachea fixed immediately post-mortem is figured as control (left column). A1. Partially decellularized tracheas (PDT) at the end of the process of decellularization, and PDT after cryopreservation at −80 °C in RPMI + 10% DMSO medium and thawing. In HES, the white arrow indicates cartilaginous fissuring. In Sirius red, the black arrows indicate the collagenous matrix, preserved after decellularization, and the dotted arrows indicate the tissue detachments observed after decellularization. In Alcian blue, black arrows indicate strong glycosaminoglycan (GAG) staining in cartilage, and dotted white arrows mark fissures, with no change in dye affinity. A2. PDT after in vivo implantation: for 28 days without immunosuppressive treatment (IS- D28), for 28 days with immunosuppressive treatment (IS+ D28), and for 56 days without immunosuppressive treatment (IS- D56). In HES, black arrows show the outer conjunctival capsule in contact with the recipient muscle, and dotted arrows designate intra-cartilaginous fissures. In Sirius red, the black arrows indicate resorption of the detachment, filled by a new collagen matrix, the dotted arrows show a more heterogeneous cartilage coloration, and the white arrow designates its degradation by the periphery. In Alcian blue, the black arrows point to areas of reduced dye affinity, reflecting the reduced GAG load. * show the tracheal lumen. Abbreviations: e (epithelium), g (glands), sm (submucosa), c (cartilage). **(B)** Axial histological sections using light microscopy and immunostaining with anti-von Willebrand factor (vWF), anti-Alpha-SMA and anti-CD3 antibodies, as well as PECAM1 in situ hybridization. Comparative sections: native trachea fixed immediately post-mortem, PDT matured for 28 days without immunosuppressive treatment (IS- D28), PDT matured for 28 days with immunosuppressive treatment (IS+ D28), and PDT matured for 56 days without immunosuppressive treatment (IS- D56). In vWF labeling, black arrows indicate neovessels. In PECAM1 hybridization, red represents PEACAM1 mRNA hybridization, therefore endothelial cells. Blue shows DAPI staining. In anti-Alpha-SMA labeling, black arrows show myofibroblastic cell infiltration. In CD3 labeling, black arrows point to the presence of T lymphocytes in the organizing tissue. Asterisks show the tracheal lumen. **C**: Quantification of blood vessels in the implanted PDT: Tissue samples were hybridized with PECAM (CD31) primers by RNAscope technology. Images were obtained by confocal microscopy (20x objective). Quantification was performed using FIBER-ML. **D.** Comparative scanning electron microscopy analysis: native trachea fixed immediately post-mortem, PDT matured for 28 days without immunosuppressive treatment (IS- D28), PDT matured for 28 days with immunosuppressive treatment (IS+ D28), and PDT matured for 56 days without immunosuppressive treatment (IS- D56). External surface shows rich collagenous extra-cellular matrix (ECM). After maturation, luminal side exposes recipient pig cells interacting with ECM at the tracheal surface (white arrows), and cross-sections show longitudinal cartilaginous fissure (asterisks) within the cartilage. A higher level of magnification reveals cartilage ring with dense ECM and chondrocyte lacunae (dotted white arrows).

Prior to implantation, the histological characteristics of fresh and cryopreserved PDTs were compared (Fig. 2*A*). HES staining revealed a continuous and intact cartilaginous structure in the fresh PDT, whereas in the cryopreserved and thawed PDT, longitudinal, non-circumferential cracks were present within the cartilage (Fig. 2*A1*,* white arrow*).

Sirius Red staining demonstrated a high concentration of collagens in the cartilage of both fresh and cryopreserved PDTs (Fig. 2*A1*,* black arrows*). However, connective tissue disorganization was observed in both conditions, with slight detachment between different tissue layers (*dotted arrows*).

Similarly, Alcian Blue staining highlighted a high concentration of glycosaminoglycans (GAGs) in the cartilage of both fresh and cryopreserved PDTs (Fig. 2*A1*,* black arrows*). After cryopreservation, focal cartilage cracks remained visible, though no significant variation in blue staining intensity was detected (*dotted white arrows*).

##### After maturation for 28 days

Histological results were compared after 28 days of *in vivo* implantation and maturation in both groups, with and without immunosuppressants. Native porcine tracheas served as the control condition (Fig. 2*A2*).

With HES staining, a connective tissue capsule was observed surrounding the PDT at the interface with the recipient muscle. This capsule was infiltrated by fibroblasts and contained minimal mononuclear inflammatory cells (Fig. 2*A2*,* black arrows*). Host-derived fibroblastic recolonization extended toward the sub-luminal layer of the PDT. On the luminal surface, a fibrinous deposit was present, but no epithelium was observed, as expected. Importantly, there was no tissue necrosis in the recipient muscle, suggesting that the PDT did not exhibit cytotoxic effects (Supplementary Fig. 3). The tracheal rings displayed longitudinal, non-circumferential small fissures similar to those seen after cryopreservation (Fig. 2 A 10, *dotted arrows*), with minimal cellular colonization in most cases.

Sirius Red staining revealed a collagenous outer capsule, with evidence of resorption of the pre-implantation detachment (*black arrows*) and collagen secretion by host fibroblasts. The cartilage collagen matrix remained preserved after *in vivo* maturation, although heterogeneous staining patterns were observed in certain regions (Fig. 2*A2*).

Alcian Blue staining showed a less intense coloration around the periphery of cartilage fissures, suggesting a reduced glycosaminoglycan (GAG) load and, consequently, possible cartilage degeneration (Fig. 2*A2*,* black arrows*).

Overall, no significant histological differences were observed between the IS^−^ and IS^+^ groups across these staining methods.

##### After maturation for 56 days

In the PDT matured *in vivo* for 56 days, HES staining revealed a thin, non-inflammatory connective outer capsule surrounding the PDT (Fig. 2*A2*). However, cartilage fissures had progressed, extending almost circumferentially. In certain areas, these fissures were associated with connective tissue infiltration and a healing response.

Sirius Red staining of the tracheal rings showed heterogeneous collagen distribution, with signs of peripheral cartilage degradation (*white arrow*). Similarly, Alcian Blue staining highlighted visible areas of cartilage degradation, along with a lighter coloration, suggesting a reduced glycosaminoglycan (GAG) content.

Overall, these findings indicate greater cartilage damage at D56 compared to D28, with more extensive structural deterioration and evidence of ongoing remodelling processes.

### Immunohistochemical characterization of cellular infiltration and neovascularization in PDTs after *in vivo* maturation

In the native trachea, small blood vessels were mainly localized in the submucosa, as demonstrated by vWF labeling. Alpha Smooth Muscle Actin (Alpha-SMA) positive cells were primarily restricted to the periphery of blood vessels, indicating the absence of fibroblasts in the rest of the tissue. Regarding lymphocyte presence, a few CD3^+^ cells were detected (Fig. 2*B*).

In the PDT, after 28 days of *in vivo* maturation, anti-von Willebrand factor (vWF) antibody labeling demonstrated the presence of neovascularization, extending down to the sub-luminal level. This pattern was observed similarly in both IS^+^ and IS^−^ groups (Fig. 2*B*). These results were also confirmed by in situ hybridization of PECAM1 (CD31) mRNA, by RNAscope technology (Fig. 2*B*). Quantification of the images in different samples showed no significant difference between the different conditions and the native trachea (Fig. 2 C), demonstrating an efficient neovascularization.

Alpha-SMA labeling revealed extensive myofibroblastic infiltration in the PDT, significantly greater than that observed in the native trachea. This infiltration reached as far as the lumen of the PDT (*black arrows*) but notably spared the cartilage.

CD3 labeling identified the presence of lymphocytes, albeit in low numbers, within the sub-luminal connective tissue but not in the cartilage (*black arrows*). Their abundance was comparable to that observed in a native trachea, and no signs of graft rejection were detected in either treatment group.

After 56 days of in vivo maturation, vascularization (vWF) and lymphocyte infiltration (CD3) remained similar to those observed at D28. However, Alpha-SMA labeling revealed increased myofibroblast infiltration, which had now extended into the cartilage fissures, indicating ongoing tissue remodeling (Fig. 2*B*).

After 28 days of *in vivo* maturation, immunofluorescence analysis of the PDT showed no apparent differences in tissue and matrix organization between the IS^−^ and IS^+^ groups (Supplementary Fig. 1).

In both groups, labeling revealed the presence of laminin within the tracheal lumen, the chorion and at the outer part of the PDT, where it interfaces with the recipient muscle.

DAPI analysis revealed host cell infiltration within the PDT, which was similar in both treatment arms, and mainly localized to the luminal surface, the chorion and the peripheral part of the PDT (Supplementary Fig. 1).

### Ultrastructural evaluation of PDT maturation using scanning electron microscopy (SEM)

SEM analysis of in the native trachea revealed that ciliary epithelial cells were present in the lumen, as expected, and no cartilage fracture was observed (Fig. 2D, native trachea).

PDTs matured *in vivo* showed an abundant extra-cellular collagenous matrix on both the peripheral and luminal sides of the PDT, regardless of experimental conditions (Fig. 2*D*). Examination of the lumen revealed the presence of host cells interacting with their extra-cellular environment.

Regarding the cartilage, deep longitudinal fissures were observed, disrupting the continuity of its thickness. At higher magnification, the microstructure of the dense extra-cellular matrix (ECM) of the cartilage appeared to be globally preserved. Chondrocyte lacunae were mostly empty (Fig. 2*C*,* cross section*). No morphological differences were detected between the IS^−^ and IS^+^ groups at 28 days of maturation, nor when compared to PDT matured for 56 days.

#### Calcification

**Fig. 3:**
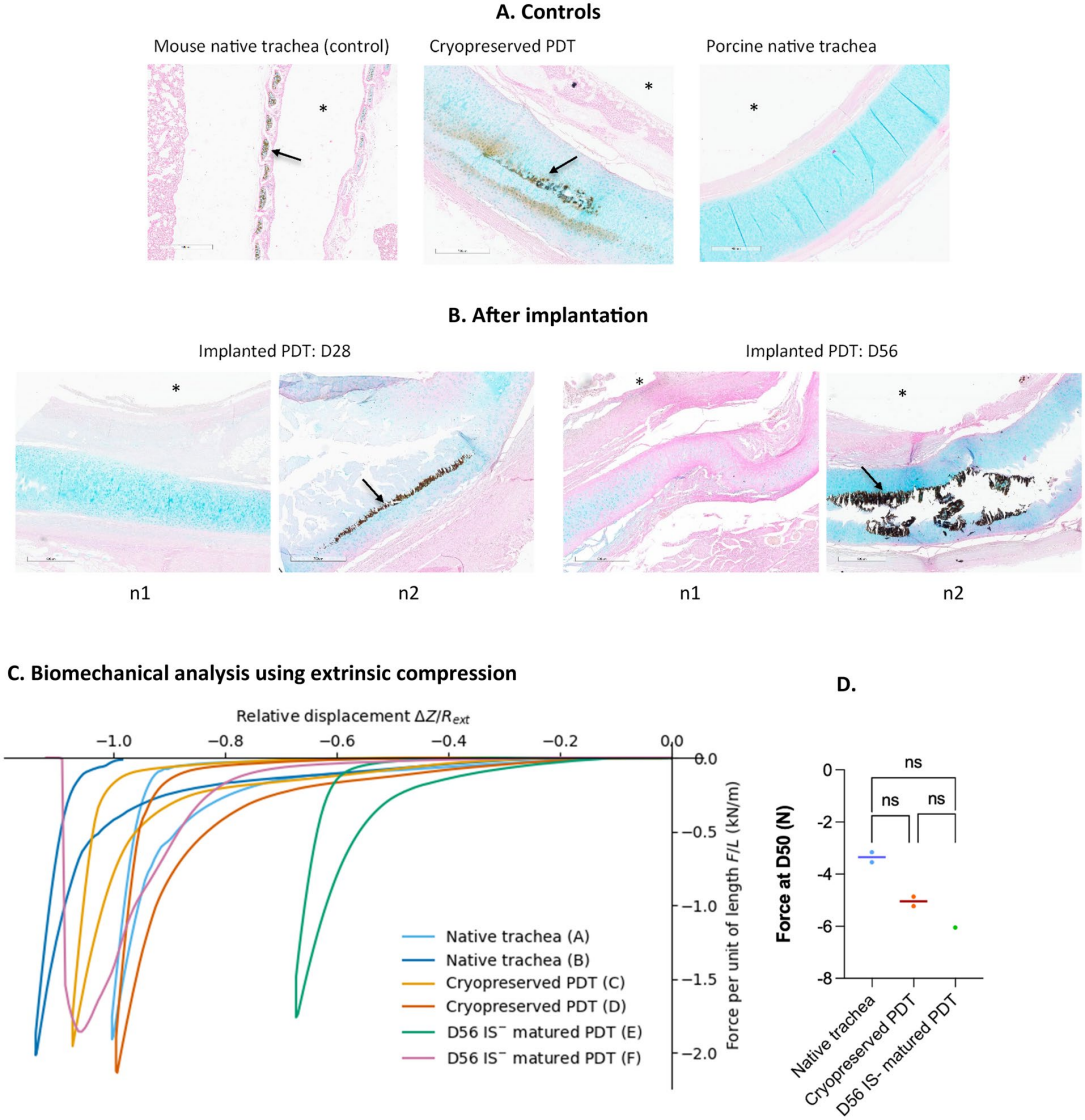
**(A)** Axial histological sections using light microscopy with von Kossa and Alcian blue staining: native trachea harvested from an implanted animal (left) cryopreserved and thawed PDT (right). Mineral deposits appear in colocalization with cartilage on cryopreserved PDT (black arrow). * show the tracheal lumen. **(B)** Axial histological sections using light microscopy with Von Kossa staining: PDT after in vivo implantation for 28 days (D28) and for 56 days (D56). For each condition, one sample showed no calcification, while the other exhibited areas of high calcification (black arrows). Asterisks show the tracheal lumen. **(C)** Radial uni-axial extrinsic compression test, on two samples of native porcine tracheae (A and B), two cryopreserved and thawed partially decellularized tracheae (PDT) (C and D), and two PDT matured in vivo for 56 days without immunosuppressant (E and F). Compression was exerted at a speed of 0.10 to 0.15 mm/second, up to a limiting force of −90 N, then unloading was carried out at the same rate. The compressive force exerted (*F*, in Newton, and by convention negative in compression) is normalized over the total length of the tracheal segment (*L*, in meters), and expressed as a function of the displacement of the crosspiece (*ΔZ*) normalized over the initial external diameter of the sample (*R*_*ext*_). **(D)** Dot plot showing the force (compressive, considered negative by convention) required to reduce tracheal lumen by 50% (D50) in native tracheas (*n* = 2), cryopreserved partially decellularized tracheas (cryopreserved PDT, *n* = 2), and in vivo matured partially decellularized tracheas after 56 days of implantation (D56 IS– matured PDT, *n* = 1, due to one other sample slippage). Each point represents an individual sample; horizontal lines show median values. There was no significant difference between groups (Kruskal-Wallis test (*p*=0.6177, *p*=0.2121 and *p*>0.9999 for native tracheas vs. cryopreserved PDT, native tracheas vs. D56 IS- matured PDT and cryopreserved PDT vs. D56 IS- matured PDT, respectively).

To identify the potential zones of calcification, von Kossa staining was performed to observe mineral deposition, while alcian blu, staining the GAGs was used to identify the cartilage. As shown in Fig. 3*A*, while staining revealed no calcification in the native trachea of an implanted pig, the cryopreserved PDT displayed calcified areas that colocalized with the cartilage, indicating mineral deposition within the scaffold. For the implanted PDTs, two animals were studied in each group (28-day and 56-day implantation, Fig. 3*B*). In both groups, one implanted PDT showed no calcification, whereas the other one showed zones of strong calcification, demonstrating heterogeneous results among samples.

### Biomechanical analyses

In order to study the effect of *in vivo* maturation on the biomechanical properties of the PDT, extrinsic compression tests were performed on two different samples of PDTs, implanted for 56 days (D56). Fresh native tracheas and cryopreserved/thawed PDTs served as controls.

These analyses revealed no differences in structural behavior between native tracheas and cryopreserved PDT (Fig. 3*C*). For the two matured PDTs (D56), *in vivo* remodeling has made sample placement more challenging, and one of these two specimens (*curve F*) slipped off the platform on the final part of the compression.

As can be seen on *curve E*, an increase in the compressive force exerted (negative, by convention) was observed for smaller platform displacements, suggesting a higher transverse rigidity of this sample matured *in vivo* (56 days). However, this was not observed with the second sample (*curve F*,* beginning of the displacement*). It is important to note that the sample corresponding to curve E had a greater parietal thickness (5.7 mm, versus 3.2 ± 0.76 mm for the others), possibly contributing to this observation. Globally, these results show no loss of transverse stiffness of PDTs after *in vivo* maturation. Statistical analysis of the results showed no significant differences between the groups (Fig. 3D).

### Systemic immune response

#### Blood analysis

##### Cyclosporin A dosages

The residual blood concentrations of cyclosporin A, analyzed at D28 in two animals in the IS^+^ cohort, were 18.6 ng/mL and 12.6 ng/mL, after 14 h from the last intake, showing the ingestion and the presence of the medication in the blood. As expected, in two animals that had not received immunosuppressive treatment, cyclosporin A was undetectable.

##### Complete blood count, hemoglobin and fibrinogen

**Fig. 4:**
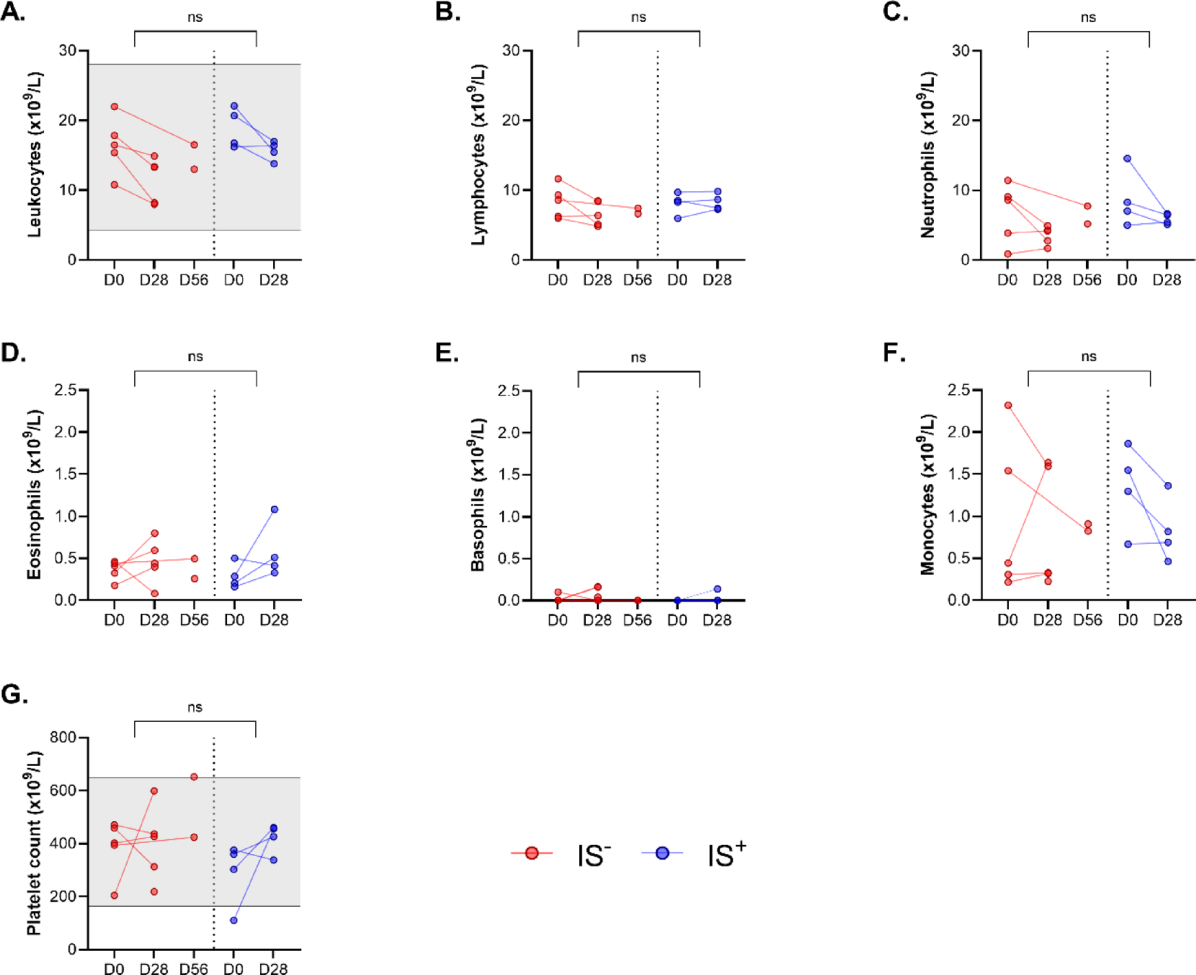
Evolution of blood cell counts in pigs between the date of implantation of the PDT (D0) and the date of explantation (D28 or D56), among the two treatment groups, without (IS-) or with (IS+) immunosuppressive treatment: leukocytes **(A)**, lymphocytes **(B)**, polymorphonuclear neutrophils **(C)**, eosinophils **(D)**, basophils **(E)**, monocytes **(F)**, and platelet count **(G)**. The gray areas represent the standards as defined by Velik-Salchner et al.

No quantitative abnormalities were observed in any of the blood cell lines across the different groups (Fig. 4).

Concerning white blood cell counts, no hyperleukocytosis was detected in either group (Fig. 4*A*). The variations in lymphocyte, monocyte, neutrophil, eosinophil, and basophil counts remained within the normal range previously described^11–13^.

Platelet counts remained within the normal range at all sampling times, except for one pig in the IS^+^ group, whose count was slightly below normal at D0 (Fig. 4*G*). Notably, individual elevations in platelets, monocytes, and eosinophils were observed in three different animals, but these variations were isolated events and did not indicate a systemic inflammatory response in any single animal.

**Fig. 5:**
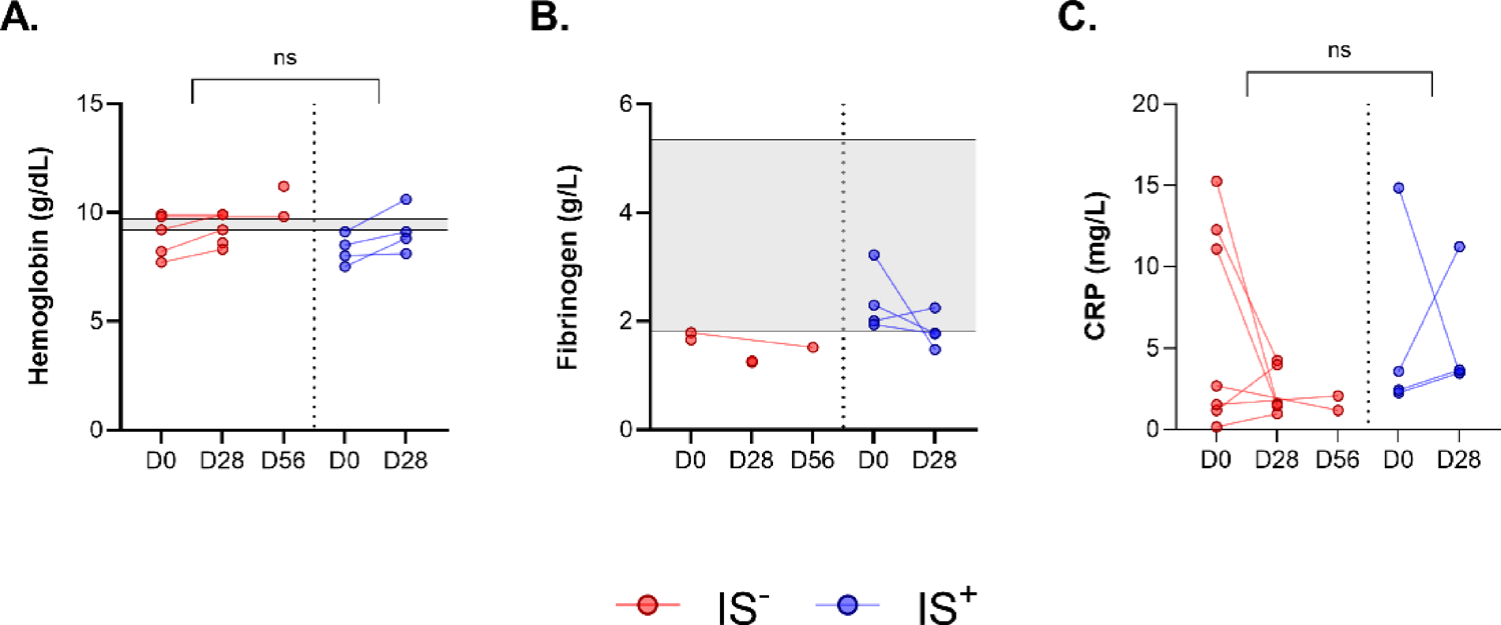
Evolution of blood protein levels in pigs between the date of implantation of the PDT (D0) and the date of explantation (D28 or D56), among the two treatment groups, without (IS-) or with (IS+) immunosuppressive treatment. The gray areas represent the standards as defined by Velik-Salchner et al. **(A)** Hemoglobin concentration on whole blood EDTA (XN analyzers). **(B)** Platelet-poor plasma fibrinogen concentration (Clauss method, STA-R analyzer). **(C)** Quantification of C-reactive protein (CRP) by ELISA technique on serum (CRP DuoSet^®^ ELISA DY2648 kit).

Blood hemoglobin levels were within the normal porcine reference range, as previously described^14^, and remained stable between implantation and explantation in both IS^+^ and IS^−^ groups. In the IS- group, the median hemoglobin level was 87 g/L [78; 97] at D0 and 92 g/L [85; 99] at D28. Similarly, in the IS+ group, hemoglobin levels measured 83 g/dL [76; 90] at D0 and 88 g/L [81; 91] at D28 (Fig. 5*A*).

Analysis of plasma fibrinogen level did not show any elevation suggestive of an inflammatory syndrome (Fig. 5*B*). At D28, the median values observed were close to the lower limit of the standards as defined by Velik-Salchner et al. (from 1.81 to 5.34 g/L): 1.26 g/L [1.24;1.27] in the IS^−^ group, and 1.78 g/L [1.55;2.13] in the IS^+^ group. These results demonstrated the absence of a systemic inflammation before and after PDT implantation.

### Serum analysis

#### C-Reactive protein (CRP)

The median of the CRP values at implantation was 11.1 mg/L [0.69;13.77] in pigs of the IS^−^ group, and 3.0 [2.32;12.04] in pigs of the IS^+^ group, without significant difference between the two groups (*p* > 0.999). Only one animal, from the IS^+^ group, presented an increase in CRP beyond 5 mg/L (11.2 mg/L) at explantation. This was the animal that presented vomiting during anesthetic induction, and had a superficial abscess of the scar.

The evolution of CRP between D0 and D28 for each animal was evaluated by the difference of the values obtained at these time points. No significant difference in evolution was highlighted between the IS^−^ group and the IS^+^ group (unpaired Mann-Whitney test, *p* = 0.4127, Fig. 5c).

##### LUMINEX (porcine specific kit)

The cytokine profile (IL-2, GM-CSF, TNF-α, IL-10, IFN-γ, IL-18/IL-1F4, IL-1RA/IL-1F3, SerpinE1/PAI-1, IL-1a/IL-1F1, IL-8/CXCL8, IL-12, IL-4, IL-6) at implantation was consistent across all animals. For all analytes, the concentration values were low, in the picogram/mL range. No significant or reproducible changes in inflammatory cytokine concentrations were observed between implantation and explantation, indicating the absence of a systemic inflammatory response. The cytokine profiles were similar in both the IS^−^ and IS^+^ groups (Table 2). 

**Table 2:**
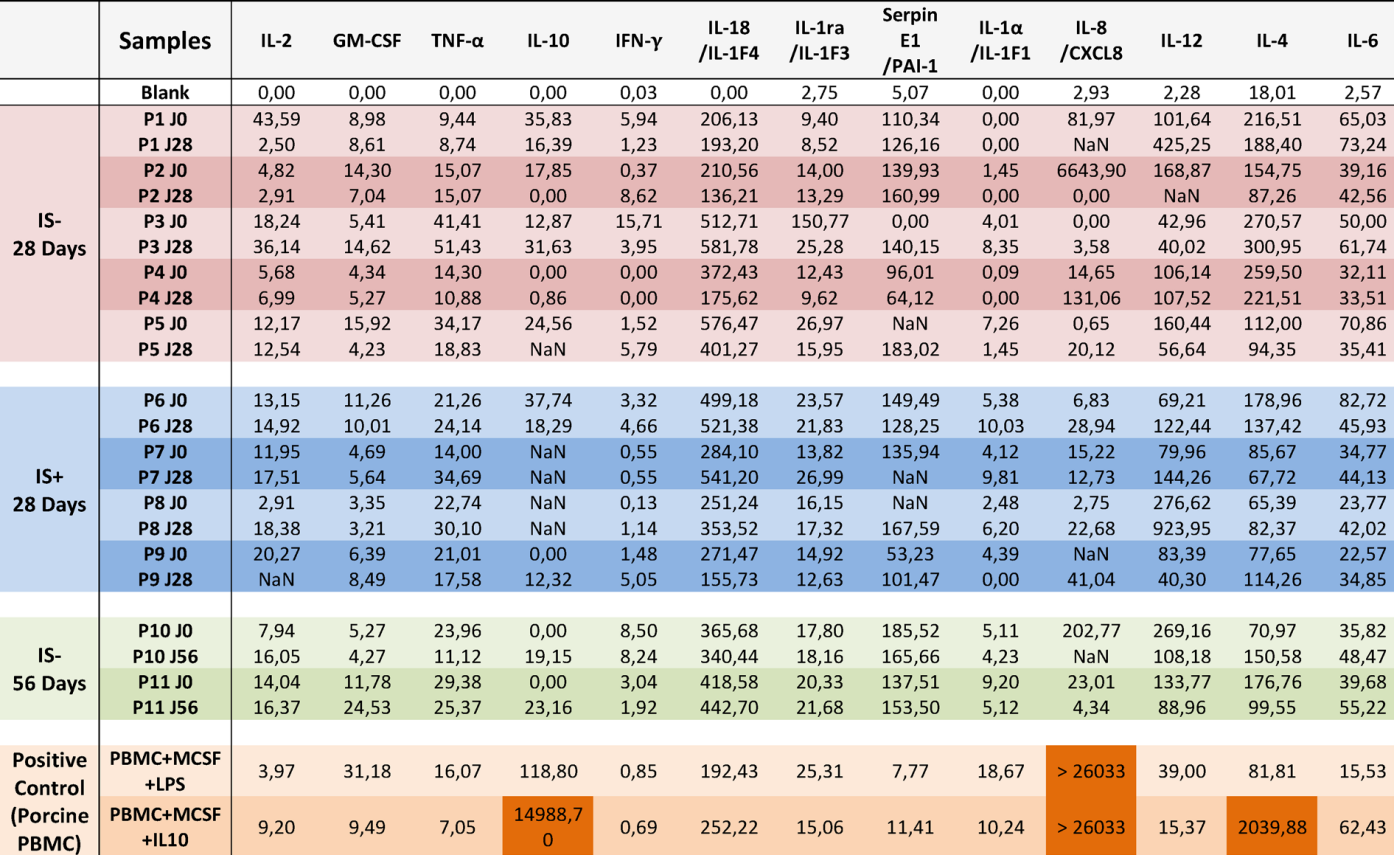
Cytokine quantification in the porcine serum by Luminex Discovery assay, using a porcine specific kit. Results are presented in pg/ml. The blank consists of Calibrator diluent used to dilute the standards. Positive controls consist of culture supernatant porcine peripheral blood mononuclear cell (PBMC), cultured in complete RPMI medium containing MCSF, supplemented with LPS or IL-10. NaN corresponds to samples that could not be analyzed due to technical errors (mainly low bead number).

## Discussion

Decellularized tissues and organs, such as the clinical-grade PDT used in this study, hold significant potential in tissue replacement and repair strategies. However, rigorous validation is required in an appropriate animal model before clinical application in humans^15^. Given its close resemblance to human tracheal anatomy and similarity in immune system function, the pig was selected as the animal model for this study^9^. This choice allows for a comprehensive evaluation of biointegration, immunological responses, biomechanical properties, and resistance to degradation.

This study of biointegration in a porcine model is the first in a series aimed at validating the PDT as a tracheal substitute. The primary objectives were evaluating the biointegration and vascularization potentials of the PDT, assessing the impact of immunosuppression on these processes, and determining the optimal maturation time before orthotopic transplantation. To sum up, this study provides a detailed analysis of the biointegration mechanisms of this type of biomaterial.

While it is widely accepted that decellularization reduces immune response and prevents graft rejection, no study to date has evaluated the effect of immunosuppression on these outcomes. In this study, cyclosporin A was chosen as the immunosuppressive agent. It is commonly used in organ transplant patients to prevent graft rejection, primarily by inhibiting T lymphocyte activation^16^.

To ensure comparability with human clinical applications while minimizing adverse effects, a dose of 3 mg/kg/day was selected all along the 28-day protocol. Cibulskyte et al. reported acute kidney failure in juvenile pigs treated with 30 mg/kg/day, resulting in euthanasia after 25 days^17^. To avoid this risk, a lower dose was chosen for this study. This dosage is consistent with initial doses recommended for organ transplant patients^18^ and the recommended doses for neonatal pigs^19^. In adult pigs, the oral dose of cyclosporin A is typically four times higher than in humans due to a larger volume of distribution, increased clearance, and lower bioavailability^20^.

In France, according to the Haute Autorité de Santé, cyclosporin A blood levels should be maintained between 100 and 300 ng/mL for organ or tissue transplant patients. Specifically, for lung transplants, the recommended range is 220–320 ng/mL in the acute phase and 140–220 ng/mL in the long term^21^. However, the cyclosporin A doses can be adjusted and reduced according to the immunological risk and the use of combination immunosuppressive therapies. In this study, cyclosporin A concentrations were below the minimum recommended thresholds but above the limit of detection, confirming treatment intake by the animals.

Comparative analysis revealed that the PDT alone did not induce graft rejection up to 56 days post-implantation, as confirmed by both local and systemic assessments. Furthermore, cyclosporin A did not enhance biointegration. These findings suggest that PDTs can be implanted without immunosuppression. This may also have broader implications for the use of other decellularized tissue grafts. Among the limitations of this study, we can mention the dosage of cyclosporin A in the blood, only at the end of the protocol. This preliminary study showed that cyclosporin A uptake by oral administration in pigs is appropriate. However, in the future studies, it is necessary to include immunosuppressive drug dosage on a clinical schedule and even consider drug injection rather than oral administration, on a larger cohort of animals.

One of the main challenges for a successful tracheal replacement is maintaining the integrity and the biomechanical properties of the cartilage rings, especially in decellularized tissues that do not contain viable chondrocytes, such as in our PDT. Even though cartilage is not immunogenic, it still undergoes in-vivo remodeling and degradation. As there are no viable chondrocytes in the PDT, the extracellular matrix (ECM) turnover and tissue homeostasis cannot be maintained^22^.Therefore, understanding the remodeling processes, and limiting damage and degradation would increase the chances of a successful tracheal replacement with PDTs.

With a perspective of a future clinical application, it is important to have a ready to use cryopreserved PDT bank. For these first in-vivo implantations, we used a simplified cryopreservation protocol, consisting of a first step at −20 °C overnight, followed by a longer preservation at −80 °C. However, the decrease in temperature was not controlled. After thawing, even before implantation, cartilage cracking along the mid-line was observed in histology, which became larger after implantation, over time. Ice crystal formation within the cartilage fragilizes its structure and causes damage and fissure. Therefore, cryopreservation, it is important to control and limit ice formation during PDT cryopreservation. As an example, cryopreservation of ovine osteochondral dowels showed that planar ice formation is very different on the surface of the cartilage compared to the intermediate spongy zone, composed of different sizes of lacunae, and more sensitive to cryopreservation. Different protocols have been tried for the cryopreservation of cartilage including higher concentrations of cryopreservative solutions such as DMSO, vitrification, as well as a cryopreservation in controlled-rate freezing systems^23,24^. In a clinical translational perspective, we have already begun the testing this latter system which has been validated for use on other tissue, by the Human Tissue Bank of St Louis hospital, Assistance Publique-Hôpitaux de Paris (APHP, Paris, France).

Another phenomenon that can alter the biomechanical properties of the tissues, mainly the cartilage, is calcification, which is due to phosphocalcic mineral deposition^25^. In our study, Von Kossa staining revealed calcification in the PDT before implantation, whereas no calcification was detected in the native trachea of the implanted animal. This could partly be explained by the difference in the age of the donor animal whose tracheas were used for the fabrication of PDT, compared to the younger receiver pig. Indeed, it has been shown that age is a major factor that plays a role in calcification of the tissues^26^ and for that same reason, our positive control was the trachea of a 21-month old mouse, which is a relatively advanced age for these animals. As for the implanted PDT, both at D28 and D56, the calcification was very heterogeneous, with one animal in each group showing strong signs of calcification, whereas the other showed none. This could also be explained by the initial state of the donors’ trachea used for PDT preparation, or heterogeneous physiological reactions. At this stage, it is difficult to conclude on the cause but longer implantation of PDT in the muscle for 56 days did not seem to increase calcification compared to 28 days. To explore our hypotheses, our future studies should include the same PDT both before and after each implantation to study the progression of calcification over time. Nevertheless, these differences did not show any major alteration of biomechanical properties, evaluated by compression tests. No weakening of the wall was observed; on the contrary, an increase in resistance was highlighted and seemed rather related to the wall thickness of the segment tested. It should be noted that in all PDTs implanted *in vivo*, no parietal thickening after maturation was observed. Despite the histological lesions observed, the extrinsic compression tests performed support the preservation of the biomechanical properties of the PDT after maturation *in vivo* (56 days).

## Conclusion

Our study has demonstrated the biocompatibility of a clinical grade PDT *in vivo*, in a heterotopic position, in a porcine model. No local or systemic inflammatory reactions nor graft rejection were observed. We confirmed the neovascularization of the PDT and its recolonization by recipient cells. Treatment with cyclosporin A did not show any additional beneficial effect on the biointegration process, compared to the untreated group. Cartilage cracks were observed after PDT cryopreservation that persisted after 28 days of *in vivo* maturation, and increased after 56 days. Within the limits of the small number of samples analyzed, no major impact on the structural biomechanical properties of PDT matured *in vivo* at 56 days was observed. To sum up, for ethical and practical reasons, this preliminary study of PDT biointegration was carried out in a small number of animals. Nevertheless, the outcomes were similar in the different groups, answering our interrogations on the first steps of biointegration, vascularization, absence of graft rejection and the absence of the necessity of immunosuppressive treatment.

In the perspective of this project, to validate the efficacy of the PDT, tracheal replacement should be pursued in an orthotopic position to study the phenomena of biointegration andreepithelialization, as well as functional respiratory efficiency. Our study identifies that 28 days without immunosuppression is the optimal condition for PDT maturation. This key finding advances our and other translational efforts for tracheal replacement in children and represents a critical step towards the development of a future clinical trial.

## Materials and methods

### Porcine tracheal harvest

The tracheas used for decellularization were from Large White/Landrace pigs, weighing between 50 and 70 kg. They were sacrificed in a professional slaughterhouse approved for research purposes, at the Institut National de Recherche pour l’Agriculture, l’Alimentation et l’Environnement (INRAe, Nouzilly, France). Tracheas were immediately transported to our laboratory in physiological saline and at room temperature.

### Partial decellularization and cryopreservation

Partially decellularized trachea (PDT) was obtained according to the methods described by Arakelian et al.^10^ and detailed in European Patent N° EP23306699,2. To summarize, the main steps of decellularization included decontamination in antibiotics overnight, SDS 1% treatment for 24 h, rinsing and removal of the residual detergent by filtration on active charcoal cartridge for 96 h, and a final step of DNase treatment and rinsing (Supplementary Fig. 2). At the end of the protocol, all cells and nuclei were removed in the soft tissue including the mucosa, submucosa, and the connective tissue. In the cartilage, cell nuclei were still present. However, no viable cells remained in the PDT.

For storage, PDTs were immersed in a 500 mL jar of Roswell Park Memorial Institute Medium (RPMI, Gibco^®^ Waltham, MA, U.S.A.) with 10% Dimethyl sulfoxide (DMSO, CryoSure-DMSO, WAK-Chemie Medical GmbH, Germany), stored overnight at −20 °C, then transferred to −80 °C. All PDTs used for implantation were cryopreserved and thawed right before the surgery.

### Microbiological analysis of PDT

In order to monitor the sterility of the PDT throughout the decellularization protocol, a sample of the tracheal tissue was isolated at each step: after decontamination, after decellularization, and before cryopreservation. A 0.5 × 1 cm full-thickness fragment of the PDT was removed using sterile instruments in a laminar flow hood, and immediately incubated in Schaedler broth (Biomérieux SA, Craponne, France) for 10 days at 37 °C. Samples were then seeded into chocolate agar + PolyViteX (Biomérieux SA) for aerobic culture, into Columbia agar + 5% sheep blood (Biomérieux SA) for anaerobic culture, and into Sabouraud agar with chloramphenicol and gentamicin agar (Bio-Rad Inc., Hercules, USA) for fungal culture. The bacteriological samples media were incubated at 37 °C for 8 days and the fungal medium at 30 °C for 11 days. Samples were also inoculated on aerobic and anaerobic BACT/ALERT^®^ flasks (Biomérieux, France) for 10 days at 37 °C. This test was performed on four different tracheas.

### *In vivo* biointegration in a porcine model

#### Summary of the experimental protocol

**Fig. 6:**
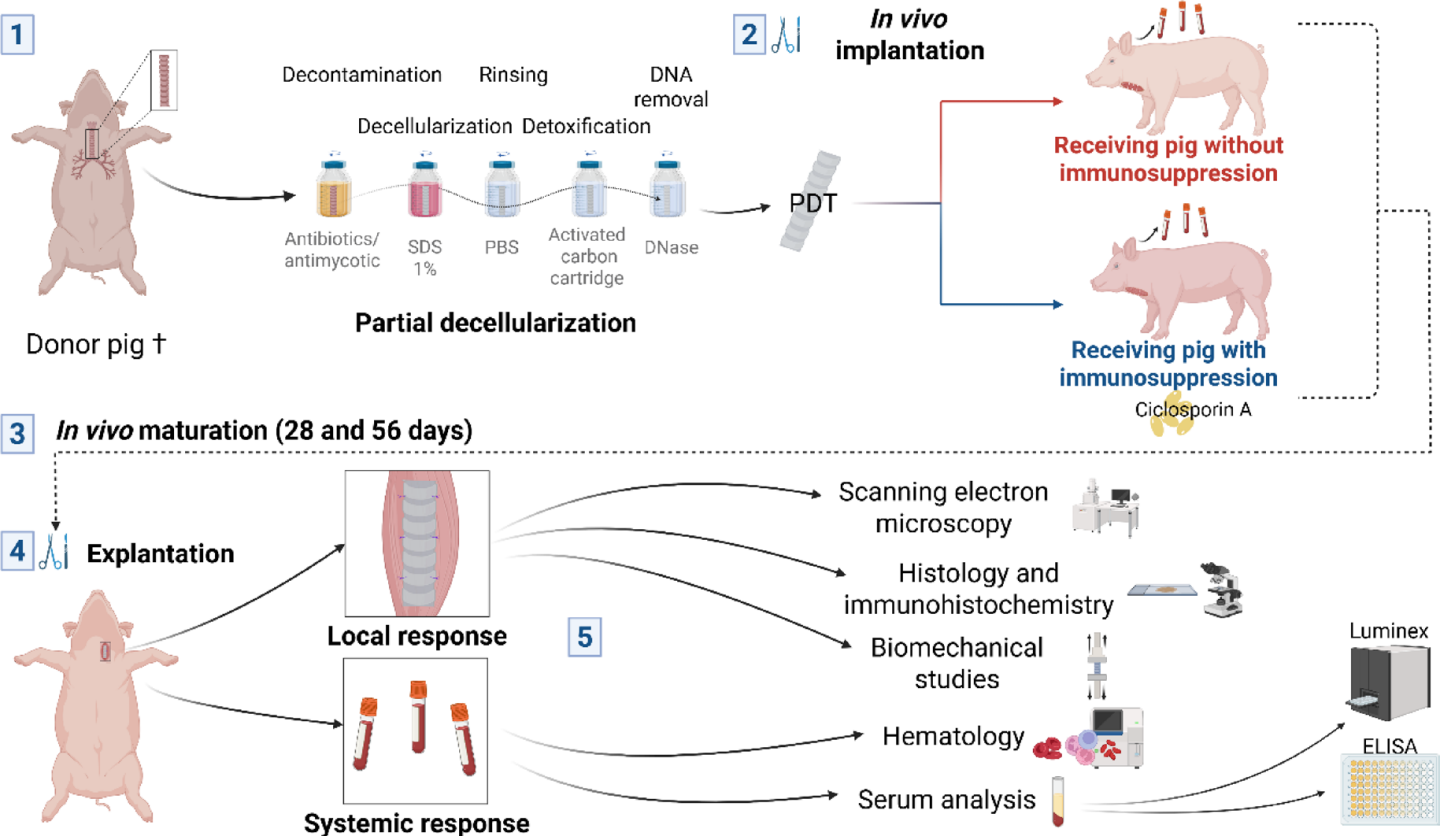
Summary of the animal experimentation protocol, from obtaining porcine cadaveric tracheas to analyzing *in vivo*-matured partially decellularized tracheae (PDT).

Eleven clinical-grade PDTs produced according to the previously described protocol (Fig. 6, *step 1*), cryopreserved and then thawed, were implanted in pigs. Animals were divided into three groups. Five animals formed the non-immunosuppressed (IS^−^) cohort with PDT maturation for 28 days, 4 animals formed the immunosuppressed (IS^+^) cohort with PDT maturation for 28 days, and 2 animals formed a third, untreated (IS^−^) group with PDT maturation for 56 days (Table 3).

**Table 3:** Summary table of animal features and surgical data at implantation and explantation.

**Animal**	**Sex**	**Group**	**Duration of heterotopic maturation**	**Length of skin incision**	**Site of implantation**	**Luminal stenting of PDT**	**Age at implantation (months)**	**Weight at implantation (kg)**	**Adverse event during implantation**	**Age at explantation (months)**	**Weight at explantation (kg)**	**Adverse event during explantation**
Pig 1	Female	IS-	28 days	145	Sterno-cephalic muscle	Rutter supra-stomal stent	5	60,0	None	6	75,0	None
Pig 2	Female	IS-	28 days	145	Sterno-cephalic muscle	Rutter supra-stomal stent	4	60,0	None	5	80,0	None
Pig 3	Female	IS-	28 days	145	Sterno-cephalic muscle	Rutter supra-stomal stent	4	60,0	None	5	80,0	None
Pig 4	Female	IS-	28 days	110	Sterno-cephalic muscle	Rutter supra-stomal stent	4	60,0	None	5	61,0	None
Pig 5	Female	IS-	28 days	105	Sterno-cephalic muscle	Rutter supra-stomal stent	4	60,0	None	5	58,6	None
Pig 6	Female	IS+	28 days	110	Sterno-hyoid muscle	Rutter supra-stomal stent	5	62,0	None	6	72,9	None
Pig 7	Female	IS+	28 days	100	Sterno-cephalic muscle	Rutter supra-stomal stent	5	62,0	None	6	67,7	None
Pig 8	Female	IS+	28 days	90	Sterno-hyoid muscle	Rutter supra-stomal stent	5	63,1	Vomiting with suspected aspiration	6	69,5	Inflamed scar with minor skin abscess
Pig 9	Female	IS+	28 days	80	Sterno-hyoid muscle	Rutter supra-stomal stent	5	59,6	None	6	67,3	None
Pig 10	Female	IS-	56 days	80	Sterno-hyoid muscle	Rutter supra-stomal stent	6	55,8	None	8	60,2	None
Pig 11	Female	IS-	56 days	70	Sterno-hyoid muscle	Rutter supra-stomal stent	6	51,1	None	8	58,5	Disinsertion of the PDT on one end of the stent

The study was conducted on female pigs, due to their calmer behavior compared to males, which facilitates monitoring and care during the implantation period. The pigs were Landrace/Large White/Pietrain hybrids. They were 4 to 5 months old at the time of the first surgery, with an average weight of 60 kg (Fig. 6, *step 2*). All animals were purchased by the Biosurgical research laboratory of Fondation Carpentier, from an accredited farm.

After a period of *in vivo* maturation (Fig. 6, *step 3*), during which the animal was monitored, a second surgical procedure involved retrieval of the PDT-recipient muscle assembly. The animal’s native trachea was also harvested after death (Fig. 6, *step 4*). Blood samples were collected at each surgery, to evaluate the systemic response. Tissue samples were divided, preserved and specifically oriented for different analyses (histology, scanning electron microscopy, immunofluorescence, biomechanics). A summary of all the treatments and studies can be found in Fig. 6, *step 5*.

All procedures and peri- and post-operative care were carried out at an accredited research facility (PARCC, Paris Cardiovascular Research Center, INSERM U970). Animal care was provided daily by animal care professionals, who corresponded with the scientific team several times a week. This research was designed and conducted in compliance with European Directives 2010/63/EU and institutional standards for animal experimentation. The experiment was approved by INSERM scientific committee, INSERM ethics committee for animal experimentation (n°INSERM-034) and the French Ministry of Education, Higher Education and Research (approval number n°2022120817007429 - V8 APAFiS # 42094). The study is reported in accordance with ARRIVE guidelines.

#### Anesthetic protocol

The surgical procedures were carried out under sterile conditions, in the animal operating room of the Laboratoire de Recherches Biochirurgicales de la Fondation Alain Carpentier (PARCC and INSERM). They were performed under general anesthetic after a 12-hour fasting period. Premedication consisted of intramuscular injection of 0.05 mg/kg acepromazine (5 mg/mL, Calmivet, Vetoquinol, Lure, France) and 15–25 mg/kg Tiletamine hydrochloride and Zolazepam hydrochloride (50 mg/mL and 50 mg/mL, Zoletil 100, Virbac, France), one hour before surgery. Anesthetic induction was achieved by intravenous injection of 4 mg/kg propofol (Proposure, Axience, France) -of which 50% was injected initially, and the remainder adapted according to effect- and 0.1 mg/kg morphine hydrochloride (Morphine Lavoisier, France). The animals were then oro-tracheally intubated and placed under controlled ventilation. Anesthesia was then continued by inhalation of 1.5 to 2.5% isoflurane on 100% oxygen, at a flow rate of 1 to 3 L/min. After induction, oxygen was delivered at 60%. Intravenous lactated Ringer’s infusion was maintained at a rate of 10–20 mL/kg/h. Antibiotic prophylaxis was administered intravenously, with 1 g (15 mg/kg) Cefazolin (Mylan, France) before skin incision, and 5 mL amoxicillin (150 mg/mL, Vetrimoxin 48 h, Ceva Santé Animale, Libourne, France) after extubation.

#### Implantation of the PDT in a pig cervical muscle

A 7–10 cm sagittal cervical skin incision was made, using the mandibular angles and sternal notch as landmarks. For the first six animals, the PDT was implanted in the left sterno-cephalic muscle^27^, along its medial edge, leaving the anterior and lateral aspects of the muscle untouched. The last five animals were implanted in the left sterno-hyoid muscle^27^, in order to minimize cervical dissection and preserve perforating vascular pedicles as far as possible. The medial edge of this muscle is immediately accessible after opening of the linea alba, and its paramedian position could subsequently offer better transposition possibilities towards the tracheal axis. A compartment was created within the chosen muscle, by longitudinal incision with a cold scalpel, in order to limit heating of the surrounding tissue. Once the muscular pocket was ready, the PDT was extended over a clinical-grade silicone stent with an external diameter of 15 mm and a length equal to that of the PDT + 15 mm (Rutter supra-stomal stent, Boston Medical Products, Shrewsbury, MA, USA), with two Ethibond 2/0 sutures (Ethicon, Johnson&Johnson France, Issy-les-Moulineaux) at each end, as described by Arakelian et al.^10^. After occluding the stent ends with a suitable plug, the PDT was inserted into the receiving muscle compartment. The muscular pouch was closed on itself by 3 to 6 stitches of 3/0 PDS (Ethicon, Johnson&Johnson France, Issy-les-Moulineaux). Vicryl 2/0 (Ethicon, Johnson&Johnson France, Issy-les-Moulineaux) was used to close the linea alba and then the skin in two planes. The scar was left uncovered for reasons of tolerance of the bandage and to facilitate clinical follow-up.

### * In vivo* maturation

The PDT was left in place *in vivo* for 28 days (D28) in 9 animals: 5 in the IS^−^ group and 4 in the IS^+^ group. To address the secondary objective of optimal maturation time, this *in vivo* maturation period was extended to 56 days for two additional animals, which did not receive immunosuppressive treatment (D56). The number of animals in each group was defined a priori by the animal research protocol that received APAFIS ministerial validation n°2,022,120,817,007,429 - V8 APAFiS # 42,094, and corresponds to all animals included until September 2024.

During the *in vivo* maturation period, animals were monitored daily (general condition, diet, body temperature, scar appearance) until PDT explantation.

#### Immunosuppressive treatment

The four animals in the IS^+^ group were placed on immunosuppressive treatment with Cyclosporin A from the day of the implantation until explantation (28 days in total). For reasons of ease of administration, team availability and cost, oral treatment was chosen. Cyclosporin A was administered in soft capsules at a dose of 3 mg/kg/day, based on human doses. With an initial mean weight of around 60 kg, each pig received 200 mg/day in two doses for 28 days. To ensure proper intake, the capsules were softened in lukewarm water and then diluted in honey.

#### PDT explantation

For the explantation, all perioperative and anesthetic procedures were similar. The surgical approach was performed based on to the initial scar. The muscular zone covering the stent-mounted PDT was harvested as a monobloc using a monopolar blade. The extremities of the stent were identified by cold instrument dissection, and the stent was removed carefully, avoiding damage to the interface between the PDT and the muscle.

The animal was sacrificed under anesthesia by intravenous injection of Pentobarbital (Doléthal, injectable solution for euthanasia, 10 mL/10 kg). After the animal was dead, the native trachea was also harvested, from the cricoid cartilage to the main bronchial divisions, as a control condition for comparative analysis.

#### Macroscopic evaluation

General and local macroscopic inspection was carried out at each surgical step. This included the general appearance and behavior of the animal, the visual aspects of the skin (scratch marks, erythema, etc.), of the surgical site and of the PDT. Any anomalies and alterations were recorded. With regard to the PDT, a maintained luminal opening was checked visually, and rigidity and flexibility were assessed subjectively by the operators. PDT measurements were taken at implantation (before and after stenting), and at explantation (before and after stent removal). Internal and external diameters and the matrix length were recorded. During the second operation, particular attention was paid to the recipient site: any signs of fibrosis, neovascularization, inflammation or surgical site infection were noted. Photographs documenting the scarring and macroscopic condition of the recipient site and the PDT before and after *in vivo* maturation were taken.

#### Blood sample collection

During each procedure (implantation and explantation), venous blood samples were taken after induction of general anesthesia. These included one to two K3-EDTA tubes (BD Vacutainer, Plymouth, UK) for complete blood count, and cyclosporin A dosage, a citrate tube (9NC BD Vacutainer, 0.129 M) for fibrinogen assay, and three to four dry tubes containing separating gel (BD Vacutainer) for serum preparation. The serum was then used for cytokine and CRP quantification.

### Biointegration evaluation

#### Histology and immunostaining

PDT or native tracheal samples were fixed in 10% formalin diluted in 1X PBS, then embedded in paraffin for histology and immunostaining.

Five micrometer-thick sections were prepared, stained with hematoxylin-eosin-safran (HES) for the evaluation of the general structure, Sirius red for collagen and Alcian blue for glycosaminoglycans (GAGs). Immunohistochemical staining was also performed using antibodies targeting CD3 (clone SP7, rabbit monoclonal antibody; Epredia Netherlands, Da Breda, Netherlands) for lymphocytes, Alpha- smooth muscle actin (Alpha-SMA, clone 1A4, mouse monoclonal antibody; Agilent Dako, Agilent Technologies France, Les Ulis) for fibroblasts and von Willebrand factor (rabbit polyclonal antibody; Agilent Dako) for endothelial cells/blood vessel detection.

#### In situ hybridization

Vascularization of PDT after implantation was also assessed by RNAscope in situ hybridization. Native tracheal and PDT samples were fixed in 10% neutral-buffered formalin and embedded in paraffin. Tissue sections at 5 μm thickness were deparaffinized with xylene and 100% ethanol. Samples were then prepared according to the manufacturer’s instructions. Endothelial cells were labelled using a swine-specific PECAM1 probe (ref. 834271-C3, Advanced Cell Diagnostics, Hayward, CA), which targets the CD31 transcript. For detection, TSA Vivid 570 fluorescent dye (Advanced Cell Diagnostics, Hayward, CA) was used. The nuclei were stained with DAPI. The slides were immediately mounted with Vectashield antifade mounting medium (Vector Laboratories, Newark, CA) and a glass coverslip. The images were acquired using an LSM 800 confocal microscope (Leica Microsystems, Wetzlar, Germany). To ensure interpretable results, the housekeeping genes POLR2A, UBC and PPIB were used as positive controls, and the bacterial DapB gene was used as a negative control.

#### PECAM (CD31) quantification

Quantification of blood vessels in the implanted PDT: Tissue samples were hybridized with PECAM (CD31) primers by RNAscope technology. Images were obtained by confocal microscopy (20x objective). Quantification was performed using FIBER-ML^28^. Label were created to characterize various type of pixels, including CD31 and Dapi staining. Then, more than 100 pixels were selected for each label, from several images. This sample was used by a machine-learning process that provided decision maps for all images. Then pixel numbers were counted for each label, for each histological image.

#### Calcification

Calcified lesions in matured PDT samples were identified by histological Von Kossa staining. Tissue sections were deparaffinized and progressively rehydrated through a series of ethanol solutions with decreasing concentrations, followed by rinsing in deionized water to prepare for staining. To detect calcification, samples were incubated for 1 h in 2% silver nitrate (Normapur, VWR) solution (prepared in deionized water; filtered) under the light of a 60-Watt lamp, washed once in ethanol 25% for 3 min, twice in deionized water for 3 min, once in 1% acetic acid for 2 min. They were then incubated for 45 min in Alcian Blue (Sigma) solution (0.02% Alcian Blue, 70% EtOH 95%, 30% acetic acid; filtered) to stain the cartilage. Samples were then washed in 1% acetic acid for 1 min, twice in deionized water for 2 min, dehydrated in successive 25%, 50% and 75% ethanol baths for 3 min each. Finally, the sections were counterstained by quick dipping in a 1% erythrosin 239 solution (Dutscher; filtered), then destained twice in 100% EtOH for 3 min, washed twice with toluene for 5 min and mounted using Pertex^®^ (Histolab).

#### Immunofluorescence

Immunofluorescence staining was performed on histological sections of PDT matured for 28 days in both IS^+^ and IS^−^ groups. After fixation in 10% formalin and embedding in paraffine, sections were deparaffinized in xylene baths and then rehydrated in baths of decreasing ethanol concentration. A rabbit polyclonal (IgG) antibody (L9393) was used to label Laminin, and revealed by anti-rabbit IgG secondary antibody coupled to Alexa Fluor 568 (Molecular Probes, A11011). DAPI was used to label nuclear DNA.

Images were acquired by wide-field fluorescence microscopy (Zeiss AxioOberver) and confocal laser scanning (Zeiss LSM 900), using a Plan-Apochromat x20/0.8 objective. Images were then processed using Zeiss Zen 3.5 and FIJI-ImageJ software.

#### Scanning electron microscopy

PDT segments intended for scanning electron microscopy (SEM) were fixed in a solution of 0.2 M Cacodylate pH 7.3 + 2.5% Glutaraldehyde + 2% Formaldehyde, and stored at 4 °C before the study. They were freed from excessive peripheral muscle tissues, using a cold blade, taking care not to alter the interface zone between the muscle and the PDT. Each sample was divided into three portions, in order to study the luminal face, the external face and the tracheal section slice in its thickness. Native trachea was also prepared as control condition.

After rinsing in cacodylate buffer to remove all traces of fixative agents, the samples were dehydrated by successive immersions in ethanol solutions of increasing concentration (30%, 50%, 80%, 100%, for 10 min for each, the last step being repeated twice). The samples were then dried by the CO_2_ critical point dryingmethod (CPD300, Leica) which allows optimal drying without destroying the structure of biological samples. Finally, the dried samples were arranged on electron microscopy stubs using carbon tape and colloidal carbon lacquer and then metallized with 5 nm of platinum (ACE600, Leica). The samples were observed under a field emission gun scanning (FEG) electron microscope (Zeiss GeminiSEM 300), in high vacuum with an accelerating voltage of 2 to 4 kV.

#### Biomechanical analyses

A biomechanical study using extrinsic compression testing was carried out to compare the material and structural strength properties between native trachea, cryopreserved PDT and *in vivo* matured PDT (Supplementary Fig. 4). Each group consisted of two samples: two PDTs cryopreserved at −80 °C in Roswell Park Memorial Institute Medium (RPMI, Gibco^®^ Waltham, MA, U.S.A.) with 10% DMSO (CryoSure-DMSO, WAK-Chemie Medical GmbH, Germany), two PDTs implanted matured *in vivo* for 56 days (D56 IS^−^), and the native tracheas of the two corresponding host animals. Biomechanical experiments were performed the day after the explantation of the matured PDT and harvesting of the native tracheas. These fresh samples were kept immersed in an antibiotic and antimycotic solution identical to that described in the decellularization protocol (gentamycin 320 mg/L, clindamycin 600 mg/L, vancomycin 500 mg/L and amphotericin B 100 mg/L) until testing. Samples were selected to be approximately 4 cm in length. They were transferred to room temperature 30 min before the mechanical test, and left in suspension in the antibiotic solution until the time of testing, performed at room temperature.

The instrument used for these experiments was manufactured at the Solid Mechanics Laboratory of the Ecole Polytechnique (LMS) and was equipped with a 100 N force cell (Instron) and a linear motor (Oriental Motor, Japan). The sample was observed via a camera (Tameron) and a telecentric lens. The whole system was controlled by LMS software, which controlled the machine’s movement, and recorded mechanical parameters (time, force, displacement) and images synchronously. Uni-axial radial compression was applied to the test specimen, placed between two metal plates attached to the test machine, with the posterior tracheal membrane at the bottom when possible. Compression was carried out at a speed of between 0.10 and 0.15 mm/s, depending on the size of the specimen: the speed was set such that the deformation was 0.5%/second. Compression was stopped at a limit force set at −90 N (N), then discharge was performed at the same rate and also recorded. The raw data were transformed to take account of the sample size. To eliminate tracheal length effects, force was divided by length (N/mm). To eliminate diameter effects, machine displacement was divided by initial tracheal diameter (dimensionless).

#### Blood analysis

##### Residual cyclosporin A in IS^+^ animals

To control cyclosporin A intake by animals, a pharmacological dosage was carried out by determination of residual cyclosporin A concentration in whole blood from two pigs in the IS^+^ group, at the end of treatment (D28). The sample was taken in the morning of explantation, after the last dose of treatment had been administered at 4 p.m. the previous day. Blood was collected in a K3-ETDA tube, and transported at room temperature to the immunology laboratory at Hôpital Necker - Enfants Malades (AP-HP) within 24 h. Cyclosporin A blood concentration was quantified by a liquid chromatography-tandem mass spectrometry (LC-MS/MS) method, recognized as the gold standard due to its accuracy and specificity. Whole blood samples were prepared by a one-step protein precipitation method. To summarize, 50 µL of calibrators, quality controls, and well-mixed pig whole blood were added into 500 µL of internal standard extracting solution (containing the internal standard D-12 Cyclosporin A and 200 µL of ZnSO4). After mix and centrifugation, 100 µL of the supernatant was analyzed on Xevo-TQD spectrometer (Waters, France) using a positive electrospray ionization (ESI) mode.

As a negative control, a cyclosporin A quantification was performed in the same conditions in two animals that had received no immunosuppressive treatment.

##### Blood cell count

Complete blood counts were performed on whole blood EDTA by XN analyzers (Sysmex, Villepinte, France) and reviewed by a senior hematologist, familiar with porcine blood cells. The parameters studied were leukocyte count (G/L) with detailed count (lymphocytes, monocytes, neutrophils, eosinophils, basophils, all in G/L), hemoglobin level (g/L), hematocrit (%), and platelet count (G/L).

##### Plasma fibrinogen

Platelet-poor plasma was obtained from citrated samples by double centrifugation at 2500 g and 20 °C for 15 min within 2 h of collection. Plasma samples were aliquoted and stored at.

−80 °C until analysis. Plasma fibrinogen (Clauss method) was measured with Dade Thrombin Reagent (Siemens Healthcare, Marburg, Germany) by STA-R analyzer (Diagnostica Stago, Asnières, France).

#### Proinflammatory cytokines in the serum

##### Serum Preparation

Blood samples at D0 and at the end of the implantation period were collected in dry tubes containing separating gel (BD Vacutainer) coagulation activator tubes. They were centrifuged for 10 min at 3000 rpm immediately after the procedure. The separated serum was then stored in 500 µL aliquots at −80 °C before cytokine quantification.

##### Cytokine quantification by LUMINEX technology

A panel of inflammatory cytokines (IL-2, GM-CSF, TNF-α, IL-10, IFN-γ, IL-18/IL-1F4, IL-1RA/IL-1F3, SerpinE1/PAI-1, IL-1a/IL-1F1, IL-8/CXCL8, IL-12, IL-4, IL-6) was analyzed on serum samples from all animals, at implantation and explantation, by Porcine Luminex^®^ Discovery Assay (Bio-Techne, Minneapolis, USA).

To obtain a positive control, previously isolated and cryopreserved porcine Peripheral Blood Mononucleated Cells (PBMC) were seeded in wells. For 3 days, they were cultured in RPMI culture medium supplemented with 10% Fetal Calf Serum (FCS) and antibiotics, in the presence of Macrophage Colony Stimulating Factor (M-CSF) at 50 ng/mL. The medium was then replaced with culture medium containing porcine interleukin-10 (IL-10) at 40 ng/mL, or lipopolysaccharide (LPS) at 10 ng/mL, for 48 h. The supernatant of these cells was collected and analyzed in LUMINEX at the same time as the samples of interest.

The plate was read by Luminex^®^ 200TM analyzer and fluorescence analyses were performed with xPonent 4.3 software.

##### CRP quantification by ELISA

Quantification of C-reactive protein (CRP) was performed on each serum sample (11 animals, at implantation and explantation), using the porcine CRP DuoSet^®^ ELISA DY2648 kit (R&D systems).

Absorbance was measured at 450 nm by VarioskanLux plate reader (Thermo Fischer Scientific, Waltham, Massachusetts, USA) and SkanIt software.

### Statistics

GraphPad (GraphPad Software Inc., USA) was used for statistical analysis. A p-value of less than 0.05 was considered statistically significant. Descriptive results are given as median and interquartile range (median [IQR]). For statistical comparisons, non-parametric Wilcoxon signed-rank tests and ANOVA test were used.

## Supplementary Information

Below is the link to the electronic supplementary material.

Supplementary Material 1

## Data Availability

Data will be made available upon request from Augustin Vigouroux augustin.vigouroux@free.fr; Lousineh Arakelian lousineh.arakelian@aphp.fr; or Briac Thierry (briac.thierry@aphp.fr).
