# Supplementary material for: Biointegration of a partially decellularized tracheal scaffold in a porcine model - preliminary results
Source: Sci Rep. 2026 Feb 21;16:10121. doi: 10.1038/s41598-026-37823-1 (PMC13022383; doi:10.1038/s41598-026-37823-1)
Supplement: Supplementary file 1 — Supplementary Material 1 [file 41598_2026_37823_MOESM1_ESM.docx]

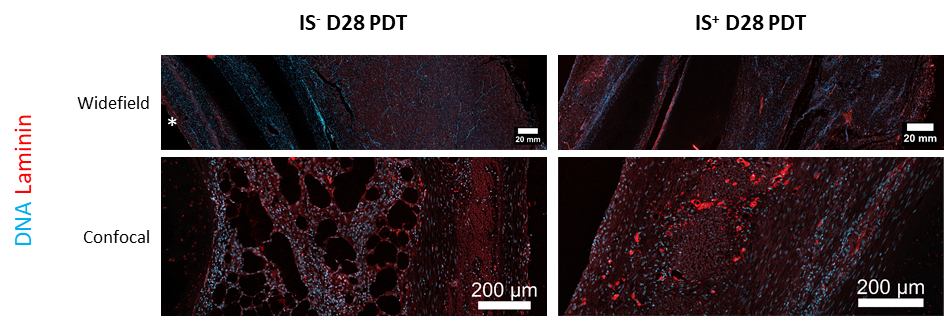


**Supplementary Figure 1:** Axial sections of partially decellularized tracheae (PDT) matured in vivo for 28 days, without (IS-) and with (IS+) immunosuppressant, observed under widefield and confocal laser scanning immunofluorescence microscopy: DAPI (blue) and laminin (red) labeling. Asterisks show the tracheal lumen.


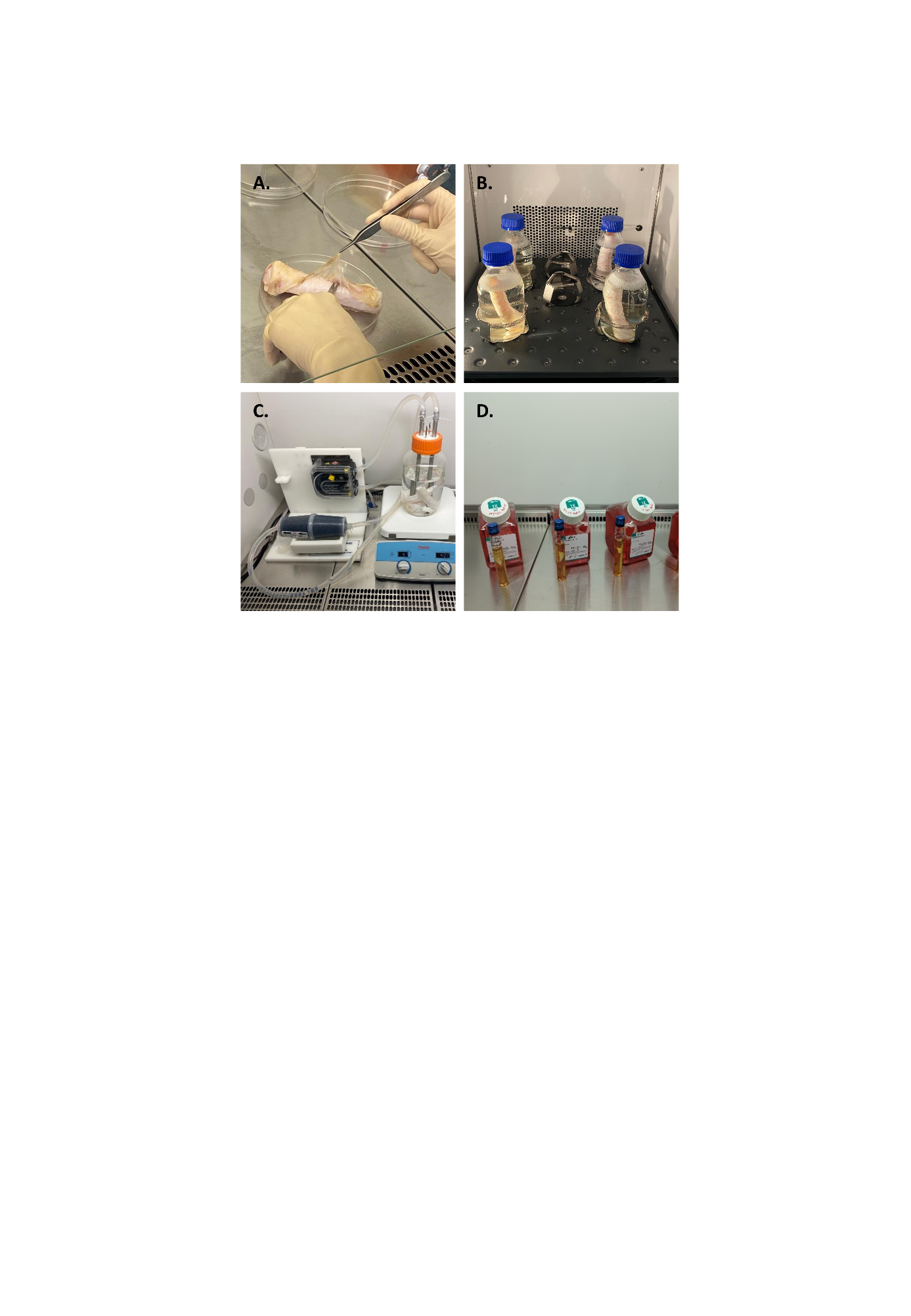


**Supplementary Figure 2:** Protocol for partial tracheal decellularization. A. Ablation of peritracheal connective tissue and adipose tissue with cold instruments, under sterile conditions in a laminar flow hood. B. Decellularization with 1% SDS under agitation at 25°C, for 24h. C. Detoxification by filtration of bath fluid on activated charcoal cartridge. D. Storage in RPMI medium + 10% DMSO, with samples in Schaedler broth tubes for sterility studies.


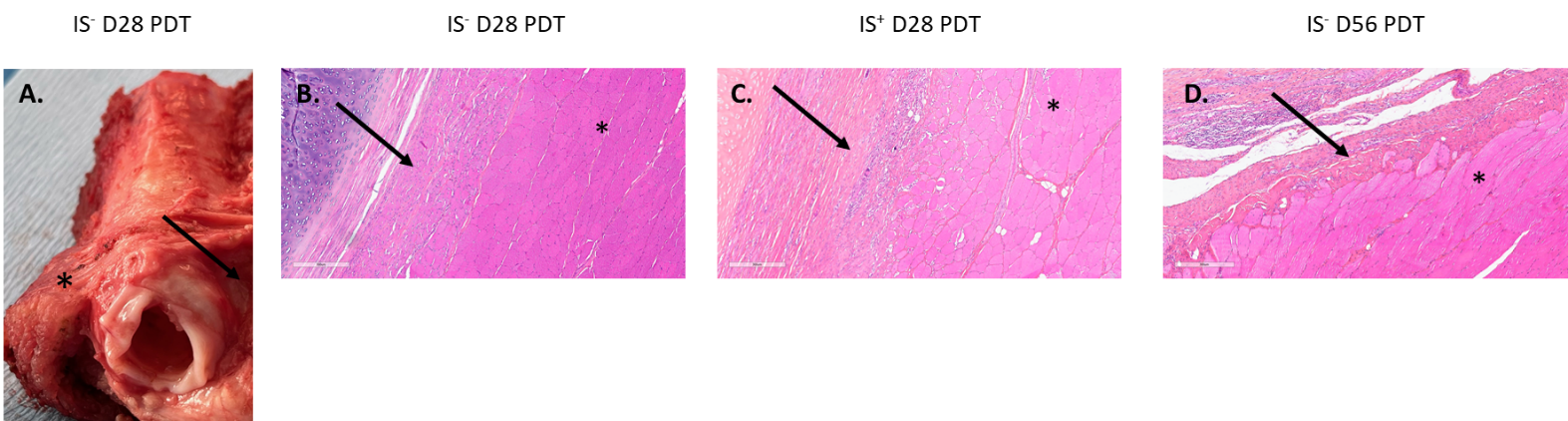


Supplementary figure 3: Macroscopic view of the implanted PDT at day 28 days without immunosuppression (A). Histological HES staining of the muscle and PDT junction at day 28 without immunosuppression (B), with cyclosporin A (B), and at day 56 without immunosuppression. The black arrows point at the muscle-PDT junction zone and the * show the surrounding muscle. Abbreviations: IS-: without immunosuppression, IS+ with immunosuppression


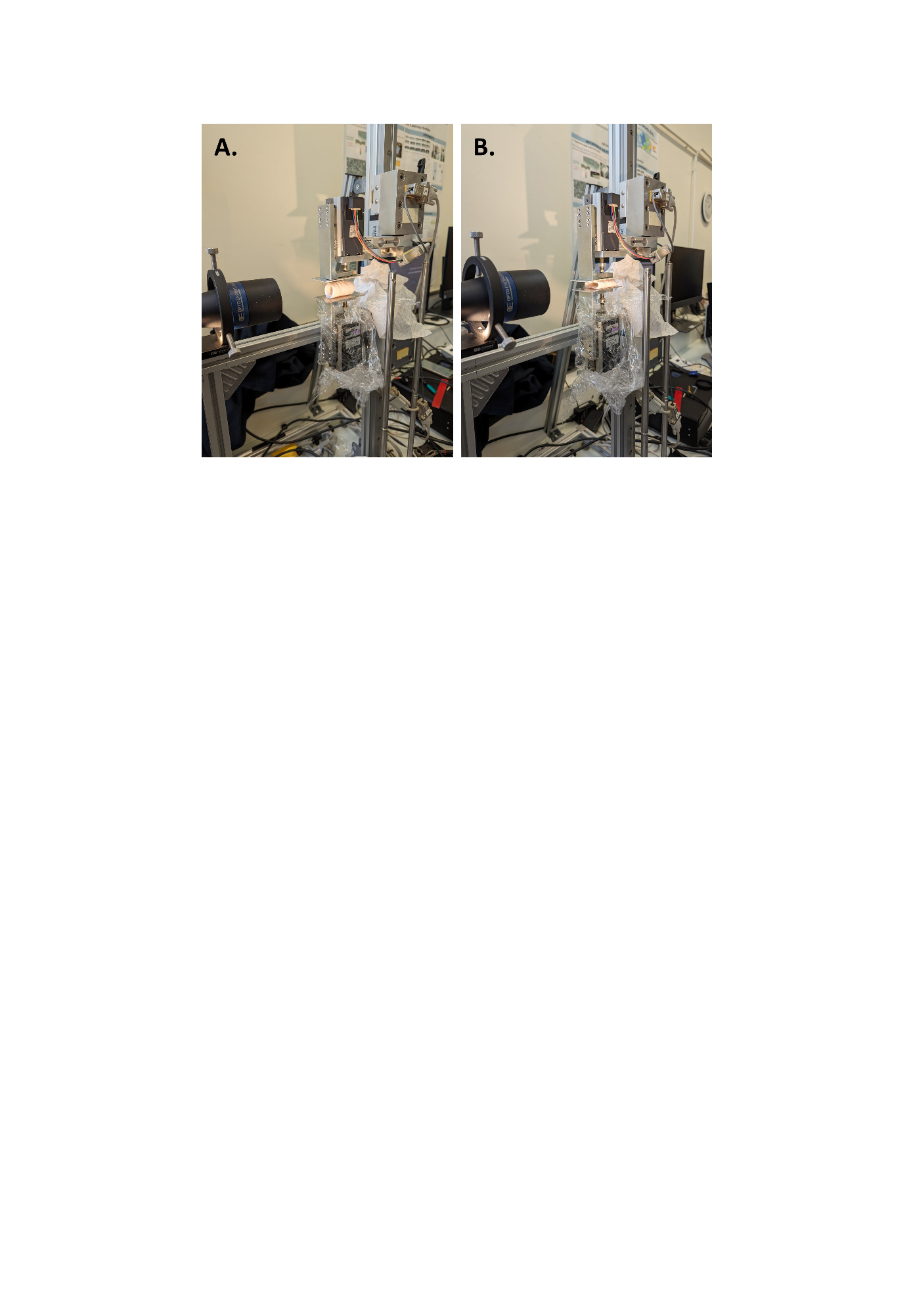


**Supplementary Figure 4:** Extrinsic radial uniaxial compression biomechanical test. The tracheal specimen is positioned, membrane down, between two metal plates attached to the testing machine (A). The compressive force is applied progressively (0.10 to 0.15mm/second, B) up to a limit set at -90 N, then lifted at the same speed. Forces and displacements are recorded live, and each test is filmed.
